# Use of period analysis to timely assess 5-year relative survival for breast cancer patients from Taizhou, Eastern China

**DOI:** 10.3389/fonc.2022.998641

**Published:** 2022-12-12

**Authors:** Runhua Li, Yabing Zheng, Jiajia Huang, Huijun Lei, Mingzhi Xu, Liangyou Wang, Luyao Zhang, Yongran Cheng, Xiyi Jiang, Huijuan Tang, Zheng Shi, Gang Chen, Huijuan Zhou, Zhijun Dai, Dalin Lu, Tianhui Chen

**Affiliations:** ^1^ Department of Cancer Prevention/Zhejiang Cancer Institute, Cancer Hospital of the University of Chinese Academy of Sciences (Zhejiang Cancer Hospital); Institute of Basic Medicine and Cancer (IBMC), Chinese Academy of Sciences, Hangzhou, China; ^2^ Department of Medical Oncology, Cancer Hospital of the University of Chinese Academy of Sciences (Zhejiang Cancer Hospital); Institute of Basic Medicine and Cancer (IBMC), Chinese Academy of Sciences, Hangzhou, China; ^3^ Department of Epidemiology, School of Medicine, Jinan University, Guangzhou, China; ^4^ Department of General Medicine, Cancer Hospital of the University of Chinese Academy of Sciences (Zhejiang Cancer Hospital); Institute of Basic Medicine and Cancer (IBMC), Chinese Academy of Sciences, Hangzhou, China; ^5^ Department of Non-communicable Chronic Disease Control and Prevention, Taizhou Center for Disease Control and Prevention, Taizhou, China; ^6^ Department of Cancer Epidemiology and Prevention, Henan Engineering Research Center of Cancer Prevention and Control, Henan International Joint Laboratory of Cancer Prevention, The Affiliated Cancer Hospital of Zhengzhou University, Henan Cancer Hospital, Zhengzhou, China; ^7^ School of Public Health, Hangzhou Medical College, Hangzhou, China; ^8^ Department of Breast Surgery, The First Affiliated Hospital, College of Medicine, Zhejiang University, Hangzhou, China; ^9^ Department of Preventive Medicine, School of Medicine, Ningbo University, Ningbo, China

**Keywords:** breast cancer, cancer registry, 5-year relative survival, period analysis, timely evaluation

## Abstract

**Objectives:**

While timely assessment of long-term survival for patients with breast cancer is essential for evaluation on early detection and screening programs, those data are extremely scant in China. We aimed to derive most up-to-date survival estimates and to predict future survival using the cancer registry data from Taizhou city, Eastern China.

**Methods:**

Patients diagnosed with breast cancer during 2004-2018 from four cancer registries with high-quality data from Taizhou, Eastern China were included. Period analysis was used to calculate 5-year relative survival (RS) for the overall population and according to the stratification factors sex, age at diagnosis and geographic region. We further predict the upcoming 5-year RS during 2019-2023, using continuous data from three 5-year periods (2004-2008, 2009-2013 and 2014-2018) and a model-based period approach.

**Results:**

Overall 6159 patients diagnosed with breast cancer during 2004-2018 were enrolled. The 5-year RS for breast cancer in 2014-2018 reached 88.8%, while women were higher compared to men (90.5% versus 83.7%) and urban areas were higher compared to rural areas (91.9% versus 86.7%). Additionally, we found a clear gradient by age at diagnosis, ranging from 94.8% for age<45 years to 83.3% for age>74 years. Projected overall 5-year RS for the upcoming 2019-2023 could reach 91.5% (84.8% for men and 93.5% for women).

**Conclusions:**

We provided, for first time in China, using period analysis, most up-to-date 5-year RS (88.8%) for patients with breast cancer from Taizhou, Eastern China. We also demonstrate the 5-year RS has improved greatly over last 15 years, which has important implications for timely evaluation of early detection and screening programs.

## Introduction

Breast cancer mainly occurs in breast epithelial tissue (1) and approximately 99% breast cancer cases arise in women (2). The burden of breast cancer continues to increase in the world (3), especially in China (4), e.g., new diagnosed cases increased from 0.30 million in 2015 to 0.42 million in 2020, and breast cancer ranked as the fourth most common cancer in China in 2020, while new death cases increased from 0.07 million to 0.12 million during 2015-2020 and ranked as the seventh leading cause of cancer-related death in 2020 (3, 5).

Long-term relative survival estimates such as 5-year relative survival are essential indicators for the assessment of cancer burden (6, 7). There is a global technical problem in assessing the long-term relative survival of cancer patients in a timely and accurate manner (8). The period analysis, first introduced in 1996 by Brenner and Gefeller (8), is the gold standard for the assessment of long-term survival of cancer patients from population-based cancer registry data and has been widely used globally (7, 9, 10). In addition, Brenner and his colleagues also proposed the model-based period analysis in 2006, which could provide a more precise survival prediction for the subsequent survival for cancer patients (11). Our group also found, for the first time systematically using period analysis and cancer registry data from Eastern China, that period analysis provides more up-to-date estimates of cancer survival compared to traditional cohort and complete methods (10). While timely assessment of long-term survival for patients with breast cancer is essential for evaluating early detection and screening programs of breast cancer, those data are extremely scarce in China.

Therefore, this article aimed to provide most up-to-date (during 2014-2018) estimates of 5-year relative survival for breast cancer patients from Taizhou, Eastern China for overall and the stratification by sex, age at diagnosis, and geographic region. Additionally, we also aimed to predict 5-year relative survival for the upcoming 2019-2023 using a model-based period analysis and the continuous survival data during 2004-2008, 2009-2013, and 2014-2018.

## Materials and methods

### Data source

Our analysis is based on data from nine cancer registries of Taizhou City between 2004–2018, and excluded cancer registries with the percent of death certificate only (DCO) above 13% from the survival analysis (10). The follow-up for breast cancer patients was completed up to 31 December 2018. Therefore, we included data from four cancer registries (Luqiao, Yuhuan, Xianju, and Wenling) for further analysis. Cancer cases were coded by the International Classification of Diseases, 10th Revision (ICD-10), and patients coded C50 were identified as patients with breast cancer. Thus, 7492 breast cancer patients were initially identified, and patients who were lost to follow-up (n = 832), unknown cases (n = 92), or missing at the last follow-up (n = 142) were excluded. After further review of the data through IARCcrgTools version 2.13 (267 cases of logic errors), 6159 eligible patients were finally included.

### Statistical analysis

Throughout this paper, relative survival (RS), which represents disease-specific survival within a patient population where cancer is the only cause of death, was used to present long-term survival estimates. The RS is derived as the ratio of observed survival rate (OSR) of the cancer patients to the expected survival of individuals corresponding with the cancer patients with similar characteristics in the calendar period of observation (12). Estimates of expected survival were derived using the Ederer II method (13) from the life-table of the four cities of Taizhou (Luqiao, Yuhuan, Xianju and Wenling) population.

In a first step, we assessed the 5-year RS of patients diagnosed in 2014–2018 by period analysis and divided patients into the newly diagnosed patients during 2014-2018 and the patients diagnosed from 2009 to 2013 but still alive within 2014-2018. The period analysis uses survival experience observed in a specified calendar period, which included left censoring of observations at the beginning of the period, while survival observations are right truncated at the end of the calendar period (14). Additionally, the method calculated the 1-year RS *Si* at the *i* year of follow-up based on the collection of a life table from cancer registries. The formula can be written as:


$$s_{i}=1-\frac{d_{i}}{n_{i}-c_{i}/2}$$

In this formula, where *n_i_* denoted the population at the beginning of the *i* year of follow-up, *d_i_* denoted the number of deaths at the end of *i* year of follow-up, and *c_i_* denoted the number of censored data in *i* year. The estimate of survival by the end of follow-up year *k* (*s_k_*) was derived from multiplying the one-year survival rate of the conditions of *k* years. The formula can be written as:


$$\bar{S_{k}}=\prod_{i=1}^{k}S_{i}$$

RS was the ratio of the observed survival divided by the expected survival. The formula was given as:


$$R_{\text{i}}=\frac{\bar{S_{k}}}{S_{k}^{*}}$$

Where $\bar{S_{k}}$ denoted observed survival, and $S_{k}^{*}$ denoted the expected survival, which is calculated using the Ederer II method. When *k*=5, the estimates of 5-year RS derived from this formula. According to the Greenwood method, the point estimate of the RS and its standard error were calculated (9).

The model-based period analysis was used to predict the 5-year RS of patients in the four counties of Taizhou City, Zhejiang Province between 2019 and 2023, with further analysis stratified by sex, age at diagnosis, and geographic region using the continuous three 5-year data during 2004-2008, 2009-2013, and 2014-2018. The 5-year RS during upcoming 2019-2023 period was then predicted by establishing a generalized linear model (GLM) *via* binomial regression with follow-up year and conditional 1-year survival rates for each year serving as independent and dependent variables (11, 15). The model-based period analysis makes full use of the data from the cancer registry system and improves the accuracy and timeliness of survival analysis (**Table 1**).

**Table 1:** Schematic diagram of the model-based period analysis.

Diagnosis year	Follow-up year			
	2004-2008	2009-2013	2014-2018	2019-2023
1999-2003				
2004-2008				
2009-2013				
2014-2018				
2019-2023				

The analyses were carried out by the package ‘period’ of R version 3.13 (R Foundation for Statistical Computing, Vienna, Austria) (16).

## Results

### Basic characteristics of breast cancer patients

The basic characteristics of the patients included in the analysis according to sex, age at diagnosis, and geographic region are presented in **Table 2**. Overall, 6159 patients diagnosed with breast cancer during 2004-2018 were included, including 6077(98.7%) females and 82(1.3%) males. The number of breast cancer cases continually increased over time (from 485 to 1814 to 3860, respectively). The average age at diagnosis was 51.3 years. The majority of patients were diagnosed with breast cancer in the 45-54 age group, with a small percentage diagnosed in the >74 age group. Moreover, the absolute number of cases was higher in rural areas (4877) than in urban areas (1282).

**Table 2:** Characteristics of breast cancer patients in four cancer registries of Taizhou City (2004-2018).

Variables	Number of cases	Diagnosed interval		
	n* (%)	2004-2008n (%)	2009-2013n (%)	2014-2018n (%)
*Gender*				
Male	82 (1.3)	9 (1.8)	33 (1.8)	40 (1.0)
Female	6077 (98.7)	476 (98.1)	1781 (98.2)	3820 (99.0)
*Geographic Region*				
Urban area	1282 (20.8)	61 (12.6)	441 (24.3)	780 (20.2)
Rural area	4877 (79.2)	424 (87.4)	1373 (75.7)	3080 (79.8)
*Age at diagnosis (years)*				
<45	1508 (24.5)	142 (29.3)	526 (29.0)	840 (21.8)
45-54	2387 (38.8)	153 (31.5)	591 (32.6)	1643 (42.6)
55-64	1391 (22.6)	74 (15.3)	411 (22.7)	906 (23.5)
65-74	618 (10.0)	61 (12.6)	192 (10.6)	365 (9.5)
>74	255 (4.1)	55 (11.3)	94 (5.1)	106 (2.7)
*Total*	6159 (100.0)	485 (100.0)	1814 (100.0)	3860 (100.0)

*n, Number of cases.

### Estimates of 5-year RS and OSR in patients with breast cancer in 2004-2018

The 5-year OSR of patients with breast cancer in 2004-2008, 2009-2013, 2014-2018 was 64.9%, 77.8%, and 83.9%, respectively, and the 5-year RS was 67.6%, 81.2%, and 88.8%, respectively (**Table 3**). Total 3860 patients were diagnosed with breast cancer from 2014 to 2018. The estimates of 5-year RS derived from the period method are presented in **Table 3**. Overall 5-year RS for breast cancer in 2014-2018 reached 88.8%, while women were higher compared to men (90.5% versus 83.7%). Additionally, we found an age gradient at diagnosis, ranging from 94.8% for age<45 years to 83.3% for age>74 years. The projected 5-year RS during 2019-2023 could reach 91.5% overall (84.8% for men and 93.5% for women). The 5-year RS in urban areas (91.9%) tended to be better than that in rural areas (86.7%).

**Table 3:** Patient survival of breast cancer by sex, age at diagnosis and geographic region in Taizhou City (2004-2018).

Variables	2004-2008		2009-2013		2014-2018	
	5-year OSR*	5-year RS	5-year OSR*	5-year RS	5-year OSR	5-year RS
*Total*	64.9	69.8	77.8	81.2	83.9	88.8
*Gender*						
Male	58.2	64.3	73.1	77.3	81.4	83.7
Female	65.2	70.2	79.3	83.8	84.9	90.5
*Age*						
<45	76.6	80.7	79.2	83.5	87.9	94.8
45-54	72.5	75.2	77.1	80.1	86.8	93.8
55-64	71.9	74.1	73.2	76.8	88.7	91.4
65-74	62.2	64.6	67.3	70.4	83.7	87.9
>74	57.1	58.6	64.1	68.8	79.2	83.3
*Region*						
Urban area	76.1	79.2	83.5	86.7	84.6	91.9
Rural area	63.4	67.7	74.5	78.6	77.2	86.7

*OSR, observed survival rate; RS, relative survival.

### The projection of 5-year RS of breast cancer patients in 2019-2023

**Figure 1:**
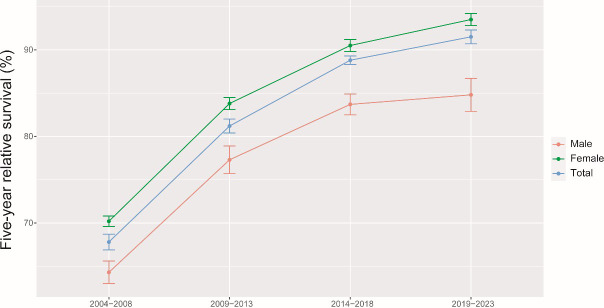
5-year relative survival of breast cancer patients by gender during 2004-2008, 2009-2013, 2014-2018 and 2019-2023.

**Figure 2:**
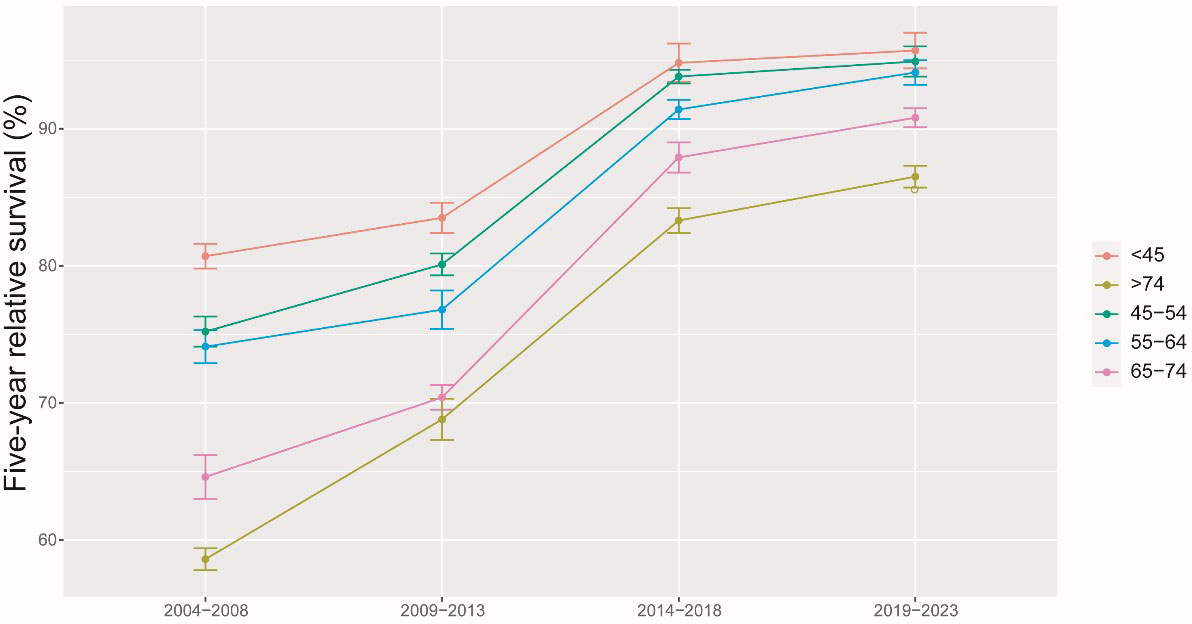
5-year relative survival of breast cancer patients by age at diagnosis during 2004-2008, 2009-2013, 2014-2018 and 2019-2023.

**Figure 3:**
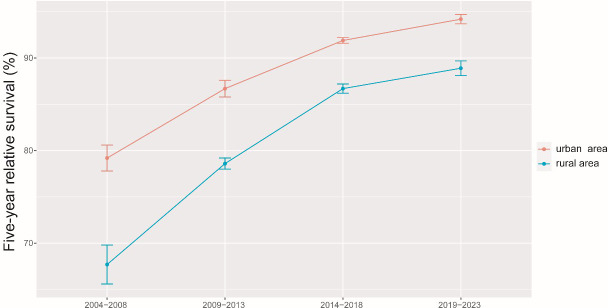
5-year relative survival of breast cancer patients by areas during 2004-2008, 2009-2013, 2014-2018 and 2019-2023.

The results of the model-based period analysis for the period of 2019-2023 are given in **Table 4**. The projected 5-year RS during 2019-2023 could reach 91.5% overall (84.8% for men and 93.5% for women). The 5-year RS for males and females during 2004-2023 shows an increasing trend, and the gap in 5-year RS between males and females was predicted to increase (**Figure 1**). Furthermore, the increasing trends were obvious in each age group between 2004 and 2023 (**Figure 2**). The gap between patients of different age groups was projected to decrease. Besides, the difference in 5-year RS between urban and rural patients gradually decreased over the period 2004-2023 (**Figure 3**).

**Table 4:** Prediction of 5-year RS* for breast cancer patients in four cancer registries of Taizhou City during 2019-2023.

Variables	Estimated value (%)
*Total*	91.5
*Gender*	
Male	84.8
Female	93.5
*Age at diagnosis (years)*	
<45	95.7
45-54	94.9
55-64	94.1
65-74	90.8
>74	86.5
*Geographic Region*	
Urban area	94.2
Rural area	88.9

*RS, relative survival.

## Discussion

In our study, we provided, for the first time systematically using period analysis in China, the most up-to-date (during 2014-2018) estimates of 5-year RS for breast cancer patients from Taizhou, Eastern China, reaching 88.8% for the overall population. While women had higher 5-year RS compared to men (90.5% versus 83.7%), urban areas had higher 5-year RS compared to rural areas (91.9% versus 86.7%). We also found a clear gradient of age at diagnosis, declining from 94.8% for age at diagnosis<45 years to 83.3% for age>74 years. We also predicted that 5-year RS for upcoming 2019-2023 could reach 91.5% for the overall population (84.8% for men and 93.5% for women). Additionally, there is a clear increasing trend in 5-year RS during 2004-2023 for the overall population and based on the stratification factors sex, age at diagnosis, and geographic region.

Breast cancer is the most common cancer among females in China (4). This is the first study systematically using period analysis to provide most up-to-date (during 2014-2018) estimates of 5-year RS for breast cancer patients from Taizhou, Eastern China, reaching 88.8% for the overall population. The previously reported RS of 82.0% during 2012-2015 were based on data from only 17 cancer registries in China (17) and was actually projected. Since cancer patients were diagnosed till the end of 2013 and followed up till the end of 2015 (17), the 5-year RS for any cancer type including breast cancer could be calculated at the latest for 2013, while it could be only projected for the survival data after 2013 and may be biased by existing data. Globally, the five-year net survival approached 90% in North America and Oceania during 2010-2014, while the survival was still low in Thailand (69%) and India (Karunagappally, 66%), possibly reflecting increased access to screening programs in wealthier countries, with subsequent impact on survival (18). Actually, from 2007 to 2017, the age-standardized death rate of breast cancer declined globally, especially in high and high-middle social-development index countries (19).

We confirmed that the survival was favorable for breast cancer patients in Taizhou City, Zhejiang Province (an overall elevation of nearly 20% points during 2004–2018), irrespective of gender, age at diagnosis and geographic region at diagnosis, although the incidence of breast cancer is still increasing, as observed in other countries (20). Firstly, the improvement in 5-year RS might be primarily attributable to the medical system reform and advances in treatment in China. Since 2009, the Ministry of Health of China launched a nationwide healthcare system reform to provide all citizens with universal medical insurance (21), which has dramatically reduced the medical financial burden to citizens. In addition, the Chinese government has gradually carried out breast cancer screening programs among women since 2008 (22). The breast cancer screening program provided additional prognostic benefits for patients (23). Furthermore, the screening has an overall estimated reduction in death from breast cancer of 28–65% (24). With the progress of medicine, the treatment has transformed from the previous single surgical treatment to a multidisciplinary comprehensive treatment. Berry and his colleagues found that the combination of screening mammography and adjuvant therapy following surgery has helped reduce the mortality from breast cancer (24). Uses of mammography, high-quality surgery, radiotherapy, and systemic therapies including chemotherapy, endocrine therapy and anti-HER2 therapy improved the survival of breast cancer patients (1).

Further analysis stratified by sex, age at diagnosis, and geographic region demonstrated substantial variations on survival rates. The observation of lower 5-year RS in male breast cancer is in line with other research results (25). Some studies showed that the less favorable outcomes in men are due to a more advanced stage at presentation, older age at diagnosis, increased comorbidities, and more aggressive tumor biology in males with breast cancer (26, 27). Moreover, the 5-year RS in males has significantly improved between 2004 and 2018. We also observed a clear age declined gradient for 5-year RS, ranging from 94.8% for age at diagnosis<45 years to 83.3% for age>74 years. Noticeably, the number of patients aged 45-54 was the largest, with an average age of diagnosis of 51.3 years old, which was similar to the results of another regional study within China (28) and the diagnosed age was lower than that in most Western countries (29). In general, the 5-year RS of all age groups at diagnosis has increased from 2004 to 2023. This could be due to improvements in treatment including adjuvant chemotherapy (30), hormonal therapy introduced in the 1980s (31), targeted therapies in the 1990s (32), and neoadjuvant chemotherapy in the 2000s (33). Additionally, we found that the 5-year RS of urban patients exceeded that of rural patients in all four periods, and the discrepancy between urban and rural areas gradually decreased between 2004 and 2023. People living in rural areas have less access to health care and facilities, and the cost is higher than for urban residents (34). Given the inequity of health service utilization and a lack of health awareness, which incurred most rural patients fail to be detected and treated in time. Delayed detection and treatment for breast cancer patients were related to poor prognosis (28). Thus, more investment for health infrastructure in underdeveloped areas, expanding medical insurance, and health education for breast cancer are urgently needed. Furthermore, the decrease in the gap between urban and rural areas might reflect the reduction in the distinction of healthcare services and the positive effect of the health system reform in China (34).

In our study, the relative survival of Taizhou City in 2014-2018 is very close to that of Shanghai Pudong (86.1%) from 2002 to 2006, while for similar period, Pudong had much higher 5-year RS compared to Taizhou (86.1% for Pudong during 2002-2006 vs. 67.6% for Taizhou during 2004-2008). These differences may be explained as follows. First, our data also included men (men had significantly lower 5-year relative survival compared to women), while Shanghai Pudong study during 2002-2006 was restricted to women. Second, Pudong New Area with all urban residents is located in Shanghai, the most developed city in China, where access to health care including breast cancer diagnosis and treatment is better than in other areas of the country. However, three (including Yuhuan, Xianju, and Wenling) out of four cancer registries in our study were from rural areas. Apparently, urban residents have better access to medical care, health education and medical insurance compared to rural residents (34–36), a fact that may have an impact on survival rates. Actually, the Shanghai Pudong report used the traditional cohort methodology (37, 38). Survival estimated by the traditional cohort approach may be underestimated, as previously shown (9, 10).

There are important differences between traditional cohort approach and period analysis approach. The traditional cohort approach uses 5-year follow-up data, delaying by at least 5 years the survival estimates (in addition to other time requests for data collection, calculation, and publication) and its survival estimate is significantly lower compared to real survival. The period analysis in our study, which does not require 5-year follow-up data to calculate survival estimates, is currently considered the “gold standard” for the assessment of the long-term survival of cancer patients using data from population-based cancer registries (9, 10).

In contrast to traditional cohort and complete methods, 5-year relative survival derived from the period analysis reflect the survival experiences of patients within the most recent period, with all patients diagnosed in the interest period. Therefore, the estimation results derived by period analysis can more accurately reflect the patients’ living conditions. Brenner et al. confirmed that the accuracy of period analysis was the best to estimate cancer survival in European cancer patients (8). Our previous study showed that the 5-year RS estimated by period analysis was the closest to the actual survival rate, and its accuracy was better than that of complete and cohort methods (10). To our knowledge, period analysis provides up-to-date survival estimates but lacks precision for the future prediction. The model-based period analysis evaluated more timely survival at much higher levels of precision (11). To acquire the latest and more accurate survival estimates for breast cancer patients and predict subsequent survival in the upcoming period, we analyzed the data based on Taizhou Cancer Registry by the model-based method. The model-based period analysis adjusted data by left censoring data diagnosed at the beginning of the interest period and right censoring data still alive at the end of the interest period. Next, we established a GLM and used the output of the regression models to carry out the subsequent survival estimates and predict the following survival trends. Given that the data for analysis included more than ten years, the predicting survival of future cancer patients using the model-based method enhanced reliability (11). Remarkably, the estimated predicted survival for breast cancer patients was up to 91.5% in 2019-2023. Other investigations also suggested of improved survival for breast cancer patients due to early detection, early diagnosis and early treatment, as well as better socioeconomic status and diet (39) (40). Besides, Taizhou City is located in East China, with a relatively constantly prosperous economy, universal medical insurance system, and continuously improving health awareness, which may continue to contribute to future improvements of breast cancer survival in Taizhou City (7, 10).

Our study has several limitations. First, detailed data on tumor stage, histological grade, hormone receptor status, and treatment modalities were unavailable in the cancer registry, so we could not conduct further analyses for outcomes. Thus, they should be assessed in future work specifically addressing this issue. Second, our study assessed the prognosis of breast cancer patients based on the indicator of 5-year RS. However, we did not calculate the longer survival since the additional data was unavailable. The investigations to assess longer-term survival for breast cancer in China are encouraged (41). Third, we evaluated long-term survival only in four groups of breast cancer patients from the population in Taizhou. Additionally, the COVID-19 epidemic might have had some impact on screening and early diagnosis of breast cancer, which may affect the precision of RS prediction in 2019-2023. And in our study, some results were based on estimates and projections; therefore, the results should be interpreted with great caution. It would be instructive to study breast cancer survival in additional geographic areas in future studies. Notwithstanding the need to take these issues into account, this study has several strengths. To our knowledge, the current investigation is the first to report the long-time survival for breast cancer patients using the period analysis and model-based period analysis based on the registry data from Taizhou City, Eastern China. In addition, we used the latest available data from Taizhou cancer registries in our analysis, the validity of cancer information and clinical data may be considered as high (7). Moreover, we further processed the data according to the rule of DCO, and the proportion of death certificates for the data was less than 13%. Thus, the results of this study were credible. This study will be a part of investigations of 5-year RS of breast cancer patients worldwide.

## Conclusions

This is the first study systematically using period analysis to provide most up-to-date (during 2014-2018) estimates of 5-year RS for breast cancer patients in Eastern China. Our study shows that the overall 5-year RS for patients with breast cancer has gradually increased, irrespective of gender, age at diagnosis and geographic region at diagnosis, which has important implication for timely evaluation of early detection and screening programs for breast cancer patients in China.

## Data availability statement

The raw data supporting the conclusions of this article will be made available by the authors, without undue reservation.

## Ethics statement

The data from nine cancer registries from Taizhou City, eastern China were completely anonymous and their use did not entail ethical problems.

## Author contributions

TC was responsible for the study concept and design. TC obtained funding. LW and TC acquired data. YC analyzed data. RL, JH, YZ, LZ, ZD, DL, and TC drafted the manuscript, and all authors revised it for important intellectual content. All authors contributed to the article and approved the submitted version.
